# Hemorrhagic shock and encephalopathy syndrome with hyper-inflammation and elevation of IL-6 and GDF-15 following COVID-19: A case report

**DOI:** 10.1016/j.idcr.2025.e02390

**Published:** 2025-10-06

**Authors:** Yoshinori Yokono, Takeshi Ebihara, Shinya Onishi, Yusuke Takahashi, Hisatake Matsumoto, Kentaro Shimizu, Satoshi Kutsuna, Jun Oda

**Affiliations:** a Department of Traumatology and Acute Critical Medicine, Graduate School of Medicine, The University of Osaka, 2-15 Yamadaoka, Suita, Osaka 565-0871, Japan; b Department of Trauma, Critical Care Medicine and Burn Center, Japan Community Health Care Organization Chukyo Hospital, 1-1-10 Sanjo, Minami-ku, Nagoya, Aichi 457-8510, Japan; c Department of Infection Control and Prevention, Osaka University Hospital, 2-2 Yamadaoka, Suita, Osaka 565-0871, Japan

**Keywords:** COVID-19, Cytokine storm, Hemorrhagic shock and encephalopathy syndrome, IL-6, Steroid therapy, Case report

## Abstract

Hemorrhagic shock and encephalopathy syndrome (HSES) is a life-threatening condition predominantly reported in children and often associated with viral infections such as influenza. We describe a case of HSES in a 21-year-old woman following Coronavirus disease-2019 (COVID-19) infection. She presented with a sore throat, nocturnal chills, and arthralgia one day before admission. On the day of admission, she developed fever and dyspnea and contacted emergency services. Upon arrival, her Glasgow Coma Scale score was E1V1M1 with tachycardia and hypotension. Tachycardia, hypotension, and lactate elevation persisted throughout the early phase of hospitalization. Orotracheal intubation and mechanical ventilation were initiated. Computed tomography revealed no intracranial lesions or pneumonia. A COVID-19 antigen test was positive, and cerebrospinal fluid analysis showed no meningitis. Despite intensive care, shock persisted with critical coagulopathy. Hemodynamic stabilization was achieved with vasopressors and fresh frozen plasma. On day 2, mydriasis developed, and head computed tomography revealed severe cerebral edema and extensive low-density areas consistent with HSES. Despite the administration of steroids and supportive care, the patient died on day 17. The patient’s interleukin-6 (IL-6) and growth differentiation factor (GDF)-15 levels were markedly elevated, with IL-6 peaking at 37,489 ng/mL and GDF-15 at 19,936 pg/mL, exceeding levels observed in other COVID-19 cases at our institution. HSES following COVID-19 infection can progress rapidly, accompanied by marked inflammatory cytokine elevation. Rapid onset of consciousness disorder, coagulopathy, and IL-6 elevation in COVID-19 or other viral infections should raise suspicion for HSES. Early recognition may aid in understanding its pathogenesis and guiding clinical care.

## Background

Coronavirus disease-2019 (COVID-19) is a severe acute respiratory syndrome coronavirus 2 (SARS-CoV-2) infection that has rapidly spread worldwide and continues to circulate with ongoing mutations. Initially presenting with upper respiratory tract symptoms, severe cases can progress to respiratory failure, circulatory failure, thromboembolism, and multiple organ dysfunction syndrome. Our previous research involving proteomics analysis of 1463 plasma proteins in COVID-19 patients identified interleukin-6 (IL-6) and growth differentiation factor-15 (GDF-15) as markers associated with disease severity and the patient prognosis [1]. Neurological symptoms related to COVID-19 increased with the emergence of the Omicron variant [2]. Hemorrhagic shock and encephalopathy syndrome (HSES) in children with COVID-19 has been reported by Sakuma et al. in 2023 [3] and Kurane et al. in 2024 [4]. However, there have been no reports on HSES caused by COVID-19 in adults. We herein report a case of HSES caused by COVID-19 in an adult patient. At the time, the predominant circulating variant of SARS-CoV-2 in Japan was Omicron sublineage XBB.

## Case presentation

A 21-year-old woman presented with a sore throat accompanied by nocturnal chills and arthralgia one day before admission. The patient had no relevant past medical history and had received two doses of the COVID-19 mRNA vaccine, but the timing of the last dose was unknown. On the morning of admission, she developed a cough and fever, for which she took over-the-counter acetaminophen. Despite taking acetaminophen, her fever and fatigue persisted into the evening. She was in contact with her family by mobile phone until she experienced difficulty breathing and called for emergency services herself. On arrival, the paramedic found the patient in a locked room, necessitating entry through a balcony. The patient was found prone on the floor, with a Glasgow Coma Scale (GCS) score of E1V1M1. Her vital signs were as follows: respiratory rate, 40 breaths/min; SpO_2_, 98 %; blood pressure, 80/30 mmHg; pulse rate, 160 beats/min; and body temperature, 40.2°C. She presented in shock, prompting intravenous access and fluid resuscitation during transport to our critical care center.

**Fig. 1:**
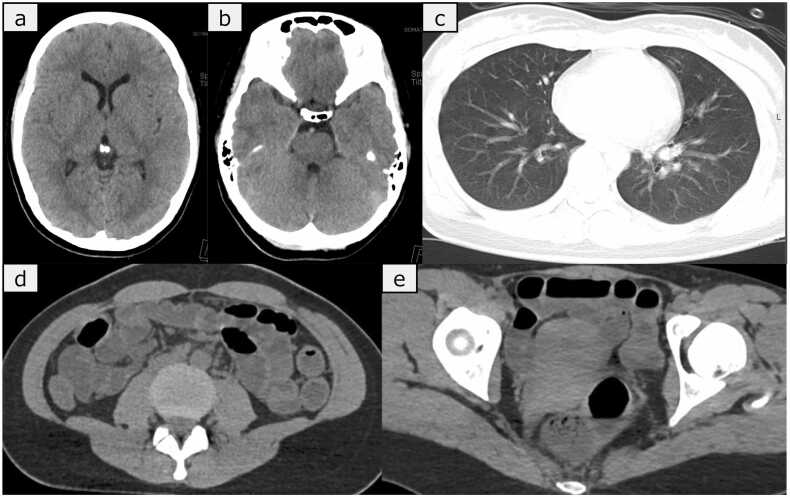
Non-contrast CT on admission. CT imaging shows no intracranial hemorrhage or hematoma in the head (a, b) and no abnormalities in the lung fields (c). Abdominal imaging reveals dilation of the small intestine with fluid accumulation but no evidence of obstruction or ascites (d) and no abnormalities in the ovaries (e).

Upon arrival at hospital, her airway was open, her breath sounds were clear, and her respiratory rate was 40 breaths/min with an SpO_2_ of 98 % on a 15 L/min non-rebreather oxygen mask. Her vital signs were as follows: blood pressure, 76/57 mmHg; heart rate, 184 beats/min; and axillary body temperature, 40.2°C. Her extremities were warm and moist with significant sweating. Abdominal ultrasound revealed a collapsed inferior vena cava and dilated small intestine. A fluid bolus was administered. Her GCS remained E1V1M1, with 4-mm pupils bilaterally reactive to light. Computed tomography (CT) showed no significant bleeding or hematoma in the brain, no pulmonary abnormalities, and a dilated small intestine without evidence of obstruction or ascites (Fig. 1). Considering seizure, particularly nonconvulsive status epilepticus (NCSE), as a differential diagnosis for her unconsciousness and tachycardia, we administered diazepam (10 mg) as a diagnostic treatment. Although the tachycardia improved, her consciousness level remained unchanged, and NCSE was subsequently ruled out. For airway management, rapid sequence intubation was performed, and mechanical ventilation was initiated. A lumbar puncture was performed to rule out meningitis, revealing colorless, clear cerebrospinal fluid (CSF) with an opening pressure of 13 cm H₂O. Gram staining and culturing were performed, but yielded no immediate evidence of bacterial infection, indicating a low likelihood of bacterial meningitis. Additionally, a multiplex PCR test using the FilmArray Meningitis/Encephalitis (ME) Panel was outsourced and performed, with results pending at that time. The details of the blood and CSF tests are shown in Table 1.

**Table 1:** At presentation, blood tests revealed thrombocytopenia and elevated D-dimer levels. Arterial blood gas analysis showed metabolic acidosis and elevated lactate levels. Cerebrospinal fluid analysis was unremarkable.

Complete blood cells			Biochemistry			Arterial blood gas analysis		
WBC	3.45	× 10 ³ /μL	Total protein	7.3	g/dL	pH	7.394	
RBC	4.66	× 10⁶/μL	Albumin	4.1	g/dL	PaO₂	26.0	mmHg
Hb	13.9	g/dL	AST	30	U/L	PaCO₂	94.7	mmHg
Hct	42	%	ALT	17	U/L	HCO₃⁻	15.8	mEq/L
Plt	12.2	× 10 ³ /μL	ALP	50	U/L	Base excess	−7.4	mEq/L
			LDH	215	U/L	SaO₂	98.3	%
Coagulation test			BUN	12	mg/dL	Lactate	54	mg/dL
PT	61	s	Cre	1.38	mg/dL	Glucose	184	mg/dL
PT-INR	1.27		eGFR	42.1	mL/min/1.73 m²			
Fibrinogen	250	mg/dL	Cl	111	mEq/L	Cerebrospinal fluid analysis		
FDP	89.4	μg/mL	K	4.2	mEq/L	Appearance	clear	
D-dimer	21.38	μg/mL	Na	144	mEq/L	Cell count	< 10	cell/μL
			CRP	2.0	mg/dL	Glucose	106	mg/dL
			NH₃	58	mg/dL	Protein	20	mg/dL

A nasopharyngeal PCR test for SARS-CoV-2 was positive. With careful attention to infection control, the patient was admitted to our hospital’s emergency intensive care unit, and intensive treatment was initiated.

Due to ongoing fluid resuscitation, the patient’s lactic acid level improved to 26 mg/dL after one hour. However, metabolic acidosis (base excess –7.4), tachycardia (heart rate, 130 bpm), and hypotension (systolic blood pressure, 90 mmHg) persisted along with watery diarrhea. At seven hours after admission, despite adequate fluid resuscitation, the patient’s mean arterial pressure dropped below 65 mmHg, and her serum lactate level increased to 34 mg/dL, meeting the criteria for septic shock. Norepinephrine was initiated at a dose of 0.1 μg/kg/min. Concurrently, hypoglycemia (blood glucose, 38 mg/dL) was observed, suggesting relative adrenal insufficiency. Based on these findings, continuous intravenous hydrocortisone was administered at a dose of 200 mg/day. Her coagulopathy worsened, with a fibrinogen level of < 35 mg/dL and her platelet count dropping to 73,000/μL. Ongoing nasal bleeding prompted the administration of 6 units of fresh frozen plasma, resulting in a fibrinogen level of 38 mg/dL, and a further 6 units increased the fibrinogen level to 104 mg/dL. Her platelet count continued to decline to 12,000/μL. While bacterial meningitis remained a possibility, the CSF findings did not strongly support it, and acyclovir was administered for suspected viral meningitis/encephalitis. Meropenem was administered empirically for bacterial infection. In addition, sudden consciousness impairment, coagulopathy, shock, and diarrhea raised concern for severe rickettsial infection, which is endemic in Japan. Therefore, levofloxacin was initially administered. Following consultation with our hospital’s infectious disease department, the antimicrobial regimen was subsequently revised to a tetracycline-based agent, which is the first-line treatment for both Japanese spotted fever and scrub typhus.

**Fig. 2:**
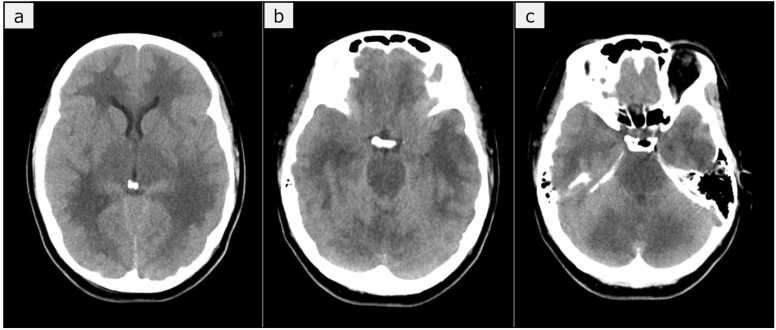
Head CT imaging 15 h after admission. The cerebrum and brainstem showed extensive low-density areas, leading to a diagnosis of diffuse brain swelling.

At 15 h after admission, the patient experienced a sudden, unanticipated elevation of her blood pressure, and her pupils dilated to 5 mm bilaterally. Given the concurrent thrombocytopenia, intracranial hemorrhage was suspected. Norepinephrine was stopped, and nicardipine was initiated for blood pressure control during a repeat head CT scan, which showed diffuse low-density areas in the cerebrum and brainstem with widespread brain swelling but no bleeding (Fig. 2).

The patient became hypotensive again. Nicardipine was stopped and norepinephrine and vasopressin were administered. Considering the presence of high fever, hypotension, thrombocytopenia, and coagulopathy, severe infection (e.g., toxic shock syndrome or rickettsial infection) was suspected, and clindamycin and minocycline were administered. Thrombotic microangiopathy was ruled out based on the absence of fragmented red cells, reticulated platelets, and low haptoglobin (54 g/dL).

Methylprednisolone (1000 mg) was administered for three days, starting 37 h after admission, as steroid pulse therapy for the acute encephalopathy. Despite treatment, the patient’s consciousness did not improve, and brainstem reflexes were absent on day 7. PCR testing of the CSF was negative for SARS-CoV-2, and the FilmArray Meningitis/Encephalitis panel detected no viral, bacterial, or fungal nucleic acids, prompting discontinuation of empiric acyclovir. Based on the clinical course and findings (including coma, shock, disseminated intravascular coagulation, elevated liver enzymes, renal dysfunction, and cerebral edema), the diagnosis of hemorrhagic shock and encephalopathy syndrome (HSES) was made, in reference to the criteria described by Levin et al. [5]

Given these findings, she did not receive escalation of care. However, due to her family’s wishes, social circumstances, and the cultural norms surrounding end-of-life care in Japan (where continued treatment is often maintained until natural death) she remained on supportive therapy until she spontaneously went into cardiac arrest and died on day 17 of hospitalization.

## Biochemical analysis of plasma and CSF

**Fig. 3:**
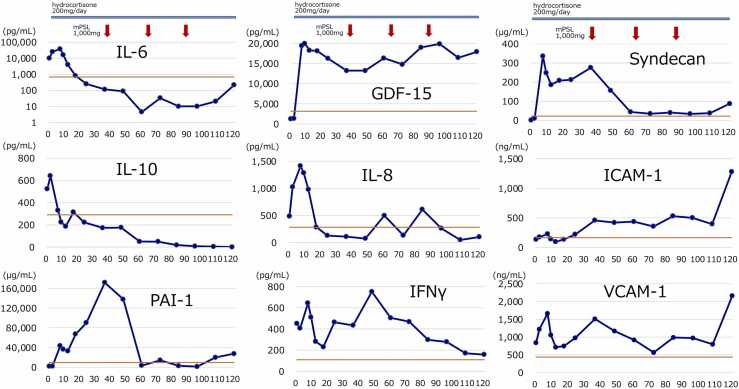
Temporal changes in biomarkers after admission. The red lines represent the average values of COVID-19 infection in cases experienced at our institution (n = 192). Hydrocortisone continuous infusion commenced from seven hours after admission, and methylprednisolone was administered at 37, 61, and 85 h after admission (red arrows). GDF-15, growth differentiation factor 15; ICAM-1, intercellular adhesion molecule 1; IFN-γ, interferon gamma; IL, interleukin; mPSL, methylprednisolone; PAI-1, plasminogen activator inhibitor 1; VCAM-1, vascular cell adhesion molecule 1.

**Fig. 4:**
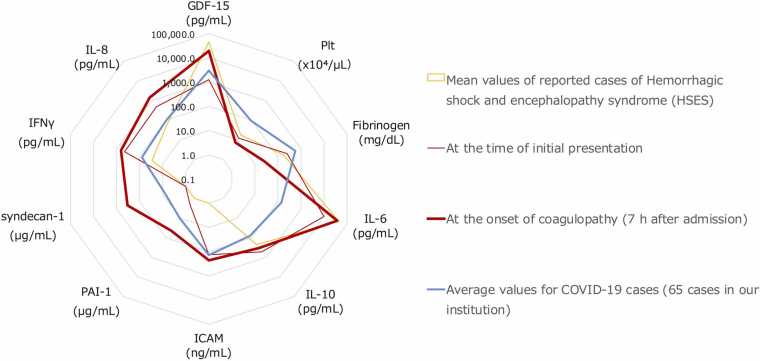
Comparison of laboratory parameters. Examination items and values measured at the initial presentation and seven hours after admission were compared with averages reported in the relevant literature on HSES and among 192 non-encephalopathic COVID-19 cases treated at our facility. Trends in cytokine elevation differed from that in non-encephalopathic COVID-19 cases but closely resembled the averages in the relevant literature. GDF-15, growth differentiation factor 15; ICAM, intercellular adhesion molecule; IFN-γ, interferon gamma; IL, interleukin; PAI-1, plasminogen activator inhibitor 1; Plt, platelets.

Plasma and CSF levels of IL-6, IL-8, IL-10, GDF-15, plasminogen activator inhibitor 1 (PAI-1), syndecan-1, intercellular adhesion molecule 1 (ICAM-1), vascular cell adhesion molecule 1 (VCAM-1), and interferon (IFN)-γ were measured by enzyme-linked immunosorbent assay (Fig. 3). At seven hours after admission, the patient’s IL-6 levels peaked at 37,489 ng/mL, and then declined. At ten hours after admission, GDF-15 reached 19,936 pg/mL, and remained elevated at 17,899 pg/mL on day 13. Endothelial damage markers ICAM-1 and VCAM-1 were elevated upon admission at 134 ng/mL and 842 ng/mL, respectively, and approximately doubled before continuous steroid administration (seven hours later). The levels decreased after continuous steroid therapy but increased again at 15 h, after the development of cerebral edema, to 458 ng/mL and 1511 ng/mL, respectively. These levels decreased again with steroid pulse therapy. Relative to the average values of HSES cases treated at our institution, previously reported HSES cases, and 65 non-encephalopathic COVID-19 patients treated at our institution [6], [7], [8], [9], [10], [11], [12], [13], [14] (Fig. 4), the values in this case and those in the reported HSES cases (shaded in gray) clearly differed from those of the non-encephalopathic COVID-19 patients (blue line), showing markedly increased levels of IL-6 and GDF-15, and decreased levels of fibrinogen and platelets.

## Discussion

This patient presented with rapid progression of decreased consciousness and coagulopathy due to COVID-19, consistent with the diagnostic criteria for HSES reported by Levin et al. [5]. The mortality rate of HSES within the acute phase (from several hours to 48 h) is reported to be 35–82 %. Many of the survivors suffered severe brain damage [15]. HSES is typically caused by viral infections such as influenza and is predominantly reported in children. Only two adult cases of HSES caused by influenza have been reported [8], [9]. Yamaguchi et al. reported initial levels of cytokines (e.g., IL-6 and GDF-15) in pediatric HSES cases associated with infection by influenza virus or of unknown etiology [6]. Although IL-6 and GDF-15 are not currently considered diagnostic markers for HSES, they have been associated with poor outcomes in COVID-19-related critical illness. Since this patient developed HSES following COVID-19 infection, we measured these biomarkers to explore their potential relevance to disease severity and pathogenesis in HSES. In the present case, the patient’s GDF-15 and IL-6 levels both increased to levels seen in previously reported HSES cases. These findings indicate that excessive cytokine production represents a common pathogenic pathway in HSES, regardless of the infecting virus. Regardless of the presence or absence of encephalopathy, it has been reported that in patients with a poor prognosis due to COVID-19, IL-6 levels initially decrease during the acute phase and then increase again on days 5–7, which was consistent with the cytokine trends observed in this case [16].

Coagulopathy rapidly developed after the admission of our patient. Continuous hydrocortisone therapy (200 mg, daily) was initiated at seven hours for sepsis, followed by steroid pulse therapy with methylprednisolone (1000 mg) at 37 h for encephalopathy. Evaluating the relationship between steroid therapy and cytokines and endothelial damage markers, the IL-6, IL-8, and IL-10 levels increased after admission and decreased after hydrocortisone administration. IFN-γ temporarily decreased after hydrocortisone administration at seven hours, then showed a rapid re-elevation, and subsequently decreased after steroid pulse therapy. Furthermore, endothelial injury markers, including ICAM-1, VCAM-1, and PAI-1, decreased following steroid pulse therapy at 37 h. This suggests that steroid therapy may contribute to the control of cytokine production and the reduction of endothelial damage.

Our patient’s GDF-15 level was 1257 pg/mL at admission, comparable to average levels in general COVID-19 cases, but it increased at seven hours after admission. GDF-15 has been reported to increase due to brain tissue damage and the elevation of inflammatory cytokines [17], [18]. It increased after vascular endothelial and brain parenchyma damage, and its elevation persisted for several days. The significant increase in GDF-15 in this case of HSES is consistent with reports linking GDF-15 to disease severity and the patient prognosis [19].

In patients with viral infections presenting with impaired consciousness, severe coagulopathy in the early stages after onset, or diarrhea from the initial phase, HSES might be considered, and the early measurement of IL-6 and GDF-15 may aid in the diagnosis. Compared with other COVID-19 cases at our institution, this patient exhibited markedly elevated cytokine levels, suggesting that hypercytokinemia may be the primary driver of the pathophysiology in HSES.

In this case, antiviral therapy for COVID-19 was not administered due to the absence of impairment of oxygenation and pulmonary abnormalities. An additional CSF analysis, including the appearance and biochemical testing, revealed no signs of infection, and a SARS-CoV-2 nucleic acid test of the CSF was negative. Antiviral therapy is generally most effective during the viral replication phase, such as within the first day of symptom onset. However, elevated cytokine levels were already present at admission, suggesting that an excessive inflammatory response may have primarily driven the rapid progression and central nervous system symptoms observed in the present case. This aligns with the current lack of clear evidence supporting the efficacy of antiviral drugs in mitigating central nervous system complications.

## Conclusions

An adult patient with HSES caused by COVID-19 showed high levels of inflammatory cytokines and endothelial damage markers in plasma and CSF. When patients with COVID-19 or other viral infections present with a rapid decrease in consciousness, coagulopathy, and elevation of IL-6, HSES should be included in the differential diagnosis. Early recognition may aid in understanding its pathogenesis and guiding clinical care.

## Author statement

None

## Author contributions

Yoshinori Yokono performed in-hospital clinical follow-up, collected specimens, performed the enzyme-linked immunosorbent assay, wrote the original article, created the figures, performed the literature review, and included the comments and corrections of the other authors. Takeshi Ebihara collected specimens, performed in-hospital clinical follow-up, and edited the manuscript. Shinya Oonishi performed the enzyme-linked immunosorbent assay and edited the manuscript. Yusuke Takahashi and Satoshi Kutsuna performed in-hospital clinical follow-up, guided treatment, and edited the manuscript. Kentaro Shimizu performed in-hospital clinical follow-up and requested a film array. Jun Oda supervised in-hospital clinical follow-up and edited the manuscript. All authors have read and approved the final manuscript.

## CRediT authorship contribution statement

**Takeshi Ebihara:** Writing – review & editing, Supervision, Project administration, Funding acquisition, Data curation, Conceptualization. **Yoshinori Yokono:** Writing – original draft, Visualization, Project administration, Methodology, Investigation, Formal analysis, Data curation, Conceptualization. **Jun Oda:** Writing – review & editing, Supervision. **Satoshi Kutsuna:** Writing – review & editing, Supervision, Methodology. **Kentaro Shimizu:** Writing – review & editing, Resources. **Hisatake Matsumoto:** Writing – review & editing, Investigation, Data curation. **Yusuke Takahashi:** Writing – review & editing, Validation, Supervision, Methodology. **Shinya Onishi:** Investigation, Formal analysis.

## Author consent

All authors have read and approved the final version of the manuscript.

This statement is submitted in accordance with the journal’s requirements for author contributions, ethical approval, and funding disclosure.

## Ethics approval and consent to participate

This study was conducted according to the principles of the Declaration of Helsinki and was approved by the institutional review board of Osaka University Hospital (Numbers: 12007, 16109 and 885 [Osaka University Critical Care Consortium Novel Omix Project; Occonomix Project]). Written informed consent was obtained from the patient for publication of this case report and accompanying images. A copy of the written consent is available for review by the Editor-in-Chief of this journal on request.

## Consent for publication

Written informed consent was obtained from the patient’s next of kin, as the patient had already died.

## Ethical approval

This study was approved by the institutional review board of Osaka University Hospital (Approval Numbers: 12007, 16109, and 885). Written informed consent for publication was obtained from the patient’s next of kin.

## Funding

This study was supported by a Grant-in-Aid for Scientific Research (KAKENHI) from the Japan Society for the Promotion of Science (JSPS) [Grant Number: JP22K09140 to TE].

## Declarations

None

## Declaration of Competing Interest

The authors declare that they have no known competing financial interests or personal relationships that could have appeared to influence the work reported in this paper.

## Data Availability

Not applicable. No datasets were generated or analyzed during the current study.

## References

[1] Ebihara T., Matsumoto H., Matsubara T., Togami Y., Nakao S., Matsuura H. (2022). Cytokine elevation in severe COVID-19 from longitudinal proteomics analysis: comparison with sepsis. Front Immunol.

[2] Stafstrom C.E. (2022). Neurological effects of COVID-19 in infants and children. Dev Med Child Neurol.

[3] Sakuma H., Takanashi J., Muramatsu K., Kondo H., Shiihara T., Suzuki M. (2023). Japanese pediatric Neuro-COVID-19 study group. Severe pediatric acute encephalopathy syndromes related to SARS-CoV-2. Front Neurosci.

[4] Kurane K., Wakae K., Yamagishi H., Kawahara Y., Ono M., Tamura D. (2024). The first case of hemorrhagic shock and encephalopathy syndrome with fulminant hypercytokinemia associated with pediatric COVID-19. Brain Dev.

[5] Levin M., Hjelm M., Kay J.D., Pincott J.R., Gould J.D., Dinwiddie R. (1983). Haemorrhagic shock and encephalopathy: a new syndrome with a high mortality in young children. Lancet.

[6] Yamaguchi H., Nishiyama M., Tokumoto S., Ishida Y., Tomioka K., Aoki K. (2021). Elevated cytokine, chemokine, and growth and differentiation factor-15 levels in hemorrhagic shock and encephalopathy syndrome: a retrospective observational study. Cytokine.

[7] Isumi H., Ichiyama T., Kawai Y., Ouchi K. (2008). Hemorrhagic shock and encephalopathy associated with rotavirus infection: a case report [in Japanese]. Infect Immun Child.

[8] Fukuda M., Yoshida T., Moroki M., Hirayu N., Nabeta M., Nakamura A. (2019). Influenza a with hemorrhagic shock and encephalopathy syndrome in an adult: a case report. Medicine.

[9] Komori Y., Uchida N., Soejima N., Fujita Y., Matsumoto H. (2020). Successful outcome in an adult patient with influenza-associated hemorrhagic shock and encephalopathy syndrome. Intern Med.

[10] Rinka H., Yoshida T., Kubota T., Tsuruwa M., Fuke A., Yoshimoto A. (2008). Hemorrhagic shock and encephalopathy syndrome--the markers for an early HSES diagnosis. BMC Pedia.

[11] Fujita Y., Imataka G., Kikuchi J., Yoshihara S. (2021). Successful mild brain hypothermia therapy followed by targeted temperature management for pediatric hemorrhagic shock and encephalopathy syndrome. Eur Rev Med Pharm Sci.

[12] Gefen R., Eshel G., Abu-Kishk I., Lahat E., Youngster I., Rosenbloom E. (2008). Hemorrhagic shock and encephalopathy syndrome: clinical course and neurological outcome. J Child Neurol.

[13] Weibley R.E., Pimentel B., Ackerman N.B. (1989). Hemorrhagic shock and encephalopathy syndrome of infants and children. Crit Care Med.

[14] Kuki I., Shiomi M., Okazaki S., Kawawaki H., Tomiwa K., Amo K. (2015). Characteristic neuroradiologic features in hemorrhagic shock and encephalopathy syndrome. J Child Neurol.

[15] Maegaki Y. (2011). Hemorrhagic shock and encephalopathy syndrome (HSES) [in Japanese. Nippon Rinsho.

[16] Domi H., Matsuura H., Kuroda M., Yoshida M., Yamamura H. (2021). Simple prognostic factors and change of inflammatory markers in patients with severe coronavirus disease 2019: a single-center observational study. Acute Med Surg.

[17] Qiu Z., Sweeney D.D., Netzeband J.G., Gruol D.L. (1998). Chronic interleukin-6 alters NMDA receptor-mediated membrane responses and enhances neurotoxicity in developing CNS neurons. J Neurosci.

[18] Schober A., Böttner M., Strelau J., Kinscherf R., Bonaterra G.A., Barth M. (2001). Expression of growth differentiation factor-15/macrophage inhibitory cytokine-1 (GDF-15/MIC-1) in the perinatal, adult, and injured rat brain. J Comp Neurol.

[19] Worthmann H., Kempf T., Widera C., Tryc A.B., Goldbecker A., Ma Y.T. (2011). Growth differentiation factor 15 plasma levels and outcome after ischemic stroke. Cereb Dis.

